# Influence of 3D-Printed TPU Properties for the Design of Elastic Products

**DOI:** 10.3390/polym13152519

**Published:** 2021-07-30

**Authors:** Lucía Rodríguez-Parada, Sergio de la Rosa, Pedro F. Mayuet

**Affiliations:** Department of Mechanical Engineering and Industrial Design, Faculty Engineering, University of Cadiz, Puerto Real, 19510 Cádiz, Spain; sergio.delarosa@uca.es

**Keywords:** flexible products, elastic products, 3D printing, additive manufacturing, TPU, industrial design, FFF, product design, customization products

## Abstract

The design of products with elastic properties is a paradigm for design engineers because the properties of the material define the correct functionality of the product. Fused filament fabrication (FFF) allows for the printing of products in thermoplastic polyurethanes (TPU). Therefore, it offers the ability to design elastic products with the freedom of forms that this technology allows and also with greater variation of elastic properties than with a conventional process. The internal structures and the variation in thickness that can be used facilitate the design of products with different elastic realities, producing variations in the elasticity of the product with the same material. This work studies the influence of the variation of internal density as a function of basic geometries in order to quantify the difference in elasticity produced on a product when it is designed. Likewise, a case study was carried out with the creation of a fully elastic computer keyboard printed in 3D. The specimens were subjected to compression to characterize the behavior of the structures. The tests showed that the elasticity varies depending on the orientation and geometry, with the highest compressive strength observed in the vertical orientation with 80% lightening. In addition, the internal lightening increases the elasticity progressively but not uniformly with respect to the solid geometry, and also the flat faces favour the reduction in elasticity. This study classifies the behavior of TPU with the aim of being applied to the design and manufacture of products with specific properties. In this work, a totally flexible and functional keyboard was designed, obtaining elasticity values that validate the study carried out.

## 1. Introduction

The design and development of new products requires long production processes, representing a large investment for the industry. In order to reduce the time in the development phases, the incorporation of additive manufacturing technologies (AM) is extended to several sectors of the industry [1,2,3].

The AM consists of a set of manufacturing technologies based on the layer-by-layer controlled deposition of the material, directly from digital product data that contain information about the geometry. The layer-by-layer manufacturing method allows for more active interaction between the properties of the final product and the manufacturing parameters. It also offers the ability to develop more complex geometries and structures with a high customization capacity, as well as a significant reduction in manufacturing time and cost. These capabilities have made AM one of the most developed technologies to date, it being very useful when low production volumes and frequent design changes are required [4,5,6].

Currently AM is showing that it can contribute effectively to technological development in the future, being the focal point of research for industries such as footwear, automotive, medical and others [7,8,9].

Part of the development of this technology over the years has also been its compatibilities with different types of materials, such as polymers, metals, ceramics, among others [10,11,12]. From this group of materials, polymers have become the center of interest due to the versatility and wide range of properties they offer. In particular, the existence of AM technologies compatible with elastomeric polymers, such as the Fused Filament Fabrication (FFF) process, has caused an increase in their use. In addition, from the perspective of designing products with flexible properties, the ability of elastomeric materials to deform and regain their shape when a force has been applied to them (elasticity) [13,14] is of great interest to different applications in industrial products and sectors such as medical, aeronautical, naval, and automobile [10,15,16].

It was found that a large number of the elastic materials compatible with the FFF process are based on Thermoplastic Polyurethane (TPU) [17,18,19,20]. One of the main reasons is that the softness of this material provides a stronger and more durable interlayer adhesion [10,15,20]. Furthermore, it is low cost and easy to use with 3D printing equipment [21]. However, the generation by FFF of substrates is a paradigm in the construction of this flexible material due to the elastic properties of the infill material and the flexibility of the filament. Very specific properties are necessary to obtain a good quality of finish. Although the manufacturers of the material provide such characteristics, there is a lack of studies in relation to the FFF process with respect to the quality of finish and functionality [22,23].

The adaptability of the design and the high customization offered by AM open up new fields of application, increasing the interest in this material for the development of products that require a certain specific elasticity. In sectors such as orthopedics and the medical field, the elasticity and the biocompatibility of TPU are especially useful for biomedical research [19,24,25]. An example of this can be the research focused on the development of fully customized soles and insoles for footwear, which aims to provide a greater degree of adaptation to the particular morphological and anthropomorphic needs of the user [14,26,27,28].

In the customization of elastic products, the study of the functional properties related to the flexibility provided by the infill material is especially relevant [21]. In this sense, FFF processes currently do not rival conventional manufacturing methods because it is possible to generate non-solid internal geometries with the possibility of reducing weight, material consumption and modify the elastic properties of the material [17]. Likewise, these internal structures offer the possibility of providing a different elasticity to the same product, as well as obtaining elastic materials from rigid materials and different elasticities from the same elastic material [29].

Under this pretext, in recent years, in the area of additive manufacturing, both companies and researchers have approached the field of products with elastic properties, increasing the research based on flexible geometric structures and solid models with a common objective of improving the functionality of these structures based on the properties of the material and the geometry [9,30,31].

A growing trend has been detected in the creating of products with deformable properties and the studying of structures that can vary the properties of the material to generate products with a certain elasticity [15,30,31,32,33]. An example of this research is the parametric study through experimentation and finite element of several designs of lattice structures carried out by Fei Shen et al. [34]. In this research, different geometric parameters are used, showing that the resistance of this type of structure varies depending on the geometric parameters used. It is demonstrated that these parameters can exert influence on the variation of the density and porosity of the structure, increasing or decreasing the reaction force under the same maximum deformation [31,35]. Another example is the research carried out by Schumacher et al. [16,36], which studies the modification of the stiffness trough the creation of multiple topologically different structures. It is possible to vary the material properties by interpolating those structures, being able to have the same behavior as a previous specified object or material.

By analyzing the literature found, the personalization has been found as a common factor, for being a field little studied and especially interesting due to its potential in several applications [37,38]. In addition, it is possible to determine that the flexible properties of TPU (and similar materials) coupled with the geometric characteristics of printed elements allow one to obtain specific properties in terms of texture, flexibility and other sensations that offer the possibility to customize the properties of elastic products.

**Figure 1:**
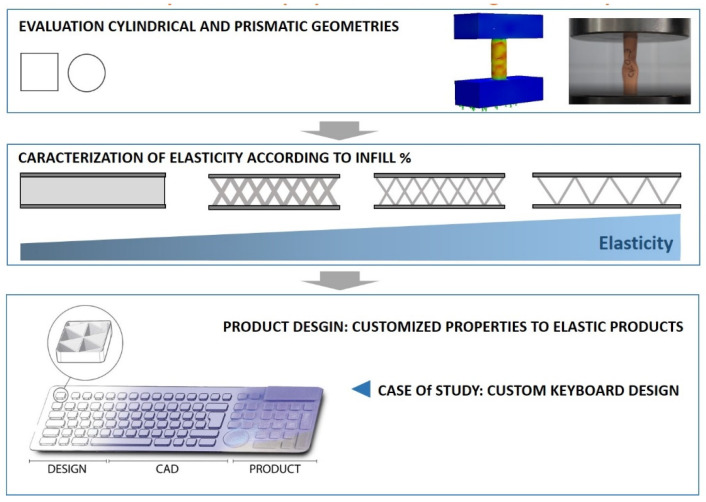
Scheme of the research and procedure for the application of research to the design of flexible or elastic products.

The studies based on consumer products, such as sports products, household goods, electronic devices, among others, are lacking and are considered to be of great interest for the design of new products with elastic properties [39,40]. In the same way, there is a lack of studies in relation to the FFF process with respect to quality of finish and functionality. Accordingly, this study focuses on evaluating the influence of geometry and internal structure on the elastic properties of the material with the objective of applying these properties to the design of custom products generated by FFF (Figure 1).

The relevance and novelty of this work lies in the creation of a working methodology that focuses on characterising the behavior of an elastic geometry that includes its own internal structure thanks to additive manufacturing. Therefore, these results serve as a basis for creating specific elastic realities adapted to the function of a specific product. They also open up a line of work to extend studies in this field and thus standardize properties of geometries with elastic materials.

For all the above-described reasons, this work was carried out in three stages: the first step in this work was the study of a total of 100 specimens, which were designed and manufactured to be measured and tested in compression. Moreover, a morphological analysis of the specimens was carried out. As a second part of the experiment, the compressive properties were studied by means of Finite Element Method (FEM) simulation. Finally, a case study was carried out with the design and development of a fully functional flexible computer keyboard.

## 2. Experimental Procedure

The experiment was proposed with the aim of characterizing the influence of geometry and internal density on the elastic properties. In addition, this study also sought to characterize the specific properties for the design and development of customised products by AM. Two types of specimens, cylindrical and prismatic, have been designed to study the elasticity with respect to the interior lightening percentage of each type of specimen.

### 2.1. Materials and Specimens

The material used in this study was the commercial Filaflex^®^ 82A TPU filament manufactured by Recreus [41]. This material has a diameter of 1.75 mm and the mechanical properties given by the manufacture are detailed in Table 1.

**Figure 2:**
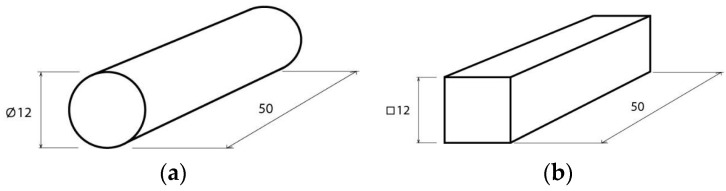
Geometry and dimensions of compression test pieces in millimetres: (**a**) cylindrical test piece and (**b**) prismatic test piece.

Using this material, two types of geometries have been studied. Type A has a circular section and Type B a prismatic section (Figure 1, respectively). These geometries were selected because they are basic geometries, from which other types of geometries can be created for product design. In addition, they were also selected in accordance with the standard UNE-EN ISO 604 [42] (Figure 2). These specimens were modeled in the Solidworks^®^ 2018 computer-aided design software.

As mentioned before, these test pieces were created to characterize the elastic properties according to the internal structure of the prototypes and products created by FFF. To this end, according to [43], several tests of resistance to compression were carried out, varying the density of the filling and the external geometry. A total of 100 specimens have been manufactured and divided into groups of 10 specimens with different properties. Five filling densities were evaluated and distributed from 0 to 100%.

Each of the 10 types of specimens has been tested in two different ways, so 10 specimens of each type have been manufactured. In accordance with [41] and [43], five specimens were used to carry out the test vertically and another five were used perpendicularly to the direction of construction. The production of the specimens was made in the Witbox^®^ model 1 cold bed printer from BQ company, using an extrusion nozzle of 0.4 mm, according to [28].

TPU material can be printed with a wide range of parameters. These parameters can be defined for the given geometrical conditions of the product to be printed [42]. In addition, the main defects with a greater influence on the final finish of the TPU product are: lack of interlayer adhesion and lack of precision and surface finish [4,23]. The parameters initially selected were defined according to [44]. For the selection of the manufacturing parameters used, a series of previous tests were carried out. The parameters used are described in the Table 2.

The production of the specimens in the 3D printer has been carried out in groups of 10 units, corresponding to each test group. The specimens have been distributed homogeneously in the printing surface. Moreover, according to [43], the specimens have been printed one by one to avoid the tensions in the models that could be generated by a simultaneous manufacturing process. The process was repeated until a total of 100 viable samples were obtained, discarding those with manufacturing defects.

On the other hand, to prepare the CAD files for the construction of the specimens and generate the STL file, the 3D printing program Ultimaker Cura V4.4 was used. In addition, by varying the fill density only, the fixed parameters used for 3D manufacturing were determined. To this end, a process optimization study was carried out to eliminate defects, which is detailed in Section 3.

The interior construction of the test pieces was carried out using an interior filling pattern with a triangular structure. The infill density was varied according to the values 100%, 80%, 50%, 20% and 0%, as shown in Table 3.

The internal structure of the filling was selected after analyzing the options provided by the Cure program.

The geometry of the filling pattern used was selected according to the investigations carried out in obtaining TPU, which show that patterns with geometries with an angle of 45° obtain better results in their mechanical properties [12], and bee cell patterns offer good results in elastic products [28,45,46]. For its application in this study, the triangular pattern was selected because it is the basic geometry that forms the bee cell type pattern. In addition, it offers better resolution and therefore less dispersion in the results due to the reduced size of the specimens. On the other hand, according to [47,48,49,50], a 45° raster angle was used in the study.

### 2.2. Procedure of Evaluation

As mentioned before, a total of 100 test tubes have been tested. The compression tests were carried out on the Shimadzu Autograph model AG-X series machine, with a maximum applicable axial force of 50 kN and a displacement resolution of 0.0208 µm. All test parameters were managed with the help of the universal software Trapezium^®^ AG-X series for Windows^®^. The compression tests were carried out in accordance with the procedure laid down in the standard [42].

It should be noted that the aim of this paper is to analyze the influence of filling parameters, as a function of their density and with a triangular pattern. Thus, compression tests were performed at a constant speed, until the breaking point of the specimen was reached. The speed was set at 1 mm/min, according to [42].

The results obtained in terms of compressive strength through the stress–strain engineering curves were analyzed, and the elastic modulus of the different specimens tested was evaluated to quantify the elasticity according to the lightening parameters studied, in accordance with [42]. Stress was obtained according to Equation (1).

(1) σ=F/S

where *F* corresponds to the axial force applied to the specimen, either in tensile or compression tests. *S* is the section area, which has been used as the actual dimension from the measurements made on the manufactured specimens.

The elastic modulus, *E*, indicates the relationship between the increase in stress and the increase in the unit longitudinal deformation that occurs during compression (Equation (2)). Then, with this equation the elasticity was quantified, increasing as the value of *E*.

(2) $$E=\frac{d\sigma }{d\varepsilon }$$

where *dσ* corresponds to the voltage variation over 5 mm and *dε* is given by Equation (3).

(3) $$\varepsilon =∆L/L_{0}$$

where *L*_0_ is the initial length of the specimen and was calculated from the average of the measurements made on the actual specimens. ∆*L* is the measurement difference between the initial distance and the distance at the measured point. The studied elastic modulus has been comprised between the displacement interval of 1 to 1.5 mm.

The evaluation of the level of elasticity was analyzed by analyzing of each of the specimens with Young’s modulus.

On the other hand, the compression tests performed were recorded by a high-precision digital camera Canon^®^ EOS 650D. The recorded images have been used to obtain the deformations of the different specimens throughout each test in order to relate them to the recorded force–displacement data.

In this type of test, these recordings are considered to be of special importance because the specimens are compressed by the elastic properties of the material and this could justify its behavior under compression. Analysis of the data obtained from the compression strength test, together with the images collected, allowed the results to be obtained in relation to the flexible properties of the tested specimens.

### 2.3. Procedure of Validation with CAE Simulation

After obtaining the experimental data during the laboratory tests, simulations of the compression tests were carried out using the Finite Element Method (FEM). Solidworks^®^ software (Dassault Systèmes S.A.) was used both for modeling the specimens and simulation.

**Figure 3:**
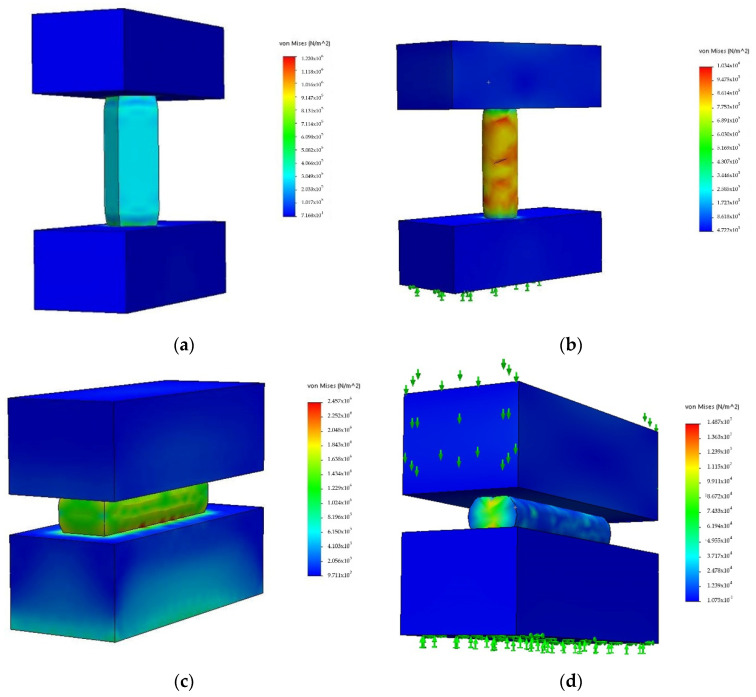
Simulation of specimen compression: (**a**) simulation of prismatic specimen in vertical orientation; (**b**) simulation of cylindrical specimen in vertical orientation; (**c**) simulation of prismatic specimen in horizontal orientation; (**d**) simulation of cylindrical specimen in horizontal orientation.

In the first place, the material of the specimen was defined. A customized elastic material has been created by introducing the properties offered by the manufacturer (Table 2). In the second place, the test boundary conditions were defined. The type of test performed is a non-linear static compression test. Both for compression, in a vertical, Figure 3a,b, and horizontal position, Figure 3c,d, it has been considered that each specimen is fixed to the ground surface by one of its flat faces. In addition, a prescribed displacement in the perpendicular direction of the opposite flat face has been imposed.

The virtual study was carried out using the vertical test pieces used to compare the simulation with the data obtained in the real tests.

## 3. Results and Discussion

The behavior of the elastic module of each specimen and the standard deviation for each model are shown in Table 4. In a first impression of the results, it is observed that the deviation of the results varies, obtaining values lower than 1, except in the case of the test carried out with the specimens PH_80 and PH_100. This can be due to small defects on the edges that cause small differences in the results between different specimens. In any case, the values are less than 1.5. According to this, in the morphological analysis, these differences will be covered in greater detail.

### 3.1. Morphological Analysis

**Figure 4:**
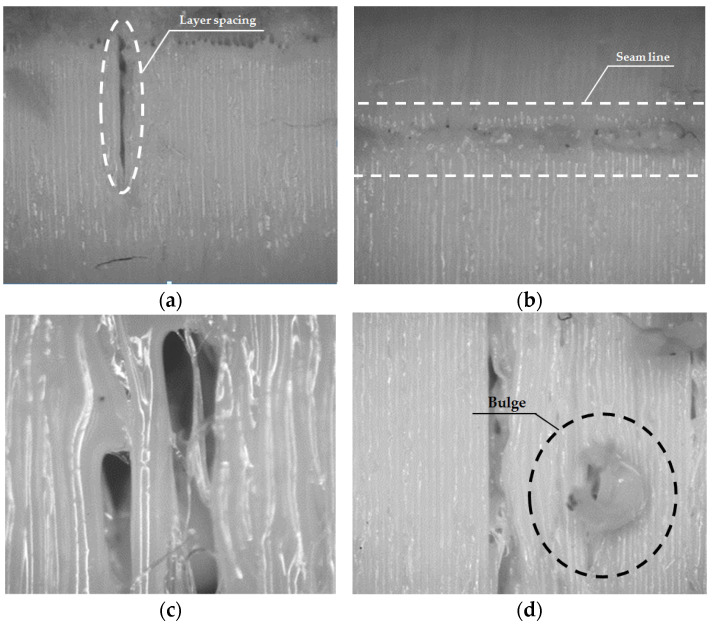
Details of manufacturing defects in specimens: (**a**) 80% filling–CH–Layer spacing; (**b**) 80% filling–CV–Seam line; (**c**) 0% filling–PH–Gap; (**d**) 20% filling–PV–Bulge.

The main defects detected in the optical microscopy analysis are shown in Figure 4. Among the most usual ones are the formation of excess material on the surface of the specimen seam and gaps on the surface. In Appendix A, Table A1 and Table A2 show in more detail the defects located on each of the manufactured specimens for prismatic and circular specimens, respectively. On the other hand, on the printing base of some of the specimens there are small defects between the layers, especially in the lighter ones [51]. Two types of defects are distinguished: discontinuities in the extrusion line and excess material [52]. However, examining the joints between layers, it is possible to observe that there are defects between one layer and another, regardless of lightening or geometry [53]. On the other hand, it is possible to highlight that specimens with 80% lightening have the best finish and the fewest defects. Finally, it should be noted that the intercalary defects are less present when the internal lightening become lower, which affects the elastic properties of the material for specimens with a low density.

### 3.2. Elasticity Analysis

Appendix B shows the behavior of each type of specimen during compression. The difference in behavior as a function of the infill density is highlighted. At low densities the geometry deforms completely, bending the outer walls. At high densities, the compressive deformation is reduced, also producing an arching of the specimen.

**Figure A1:**
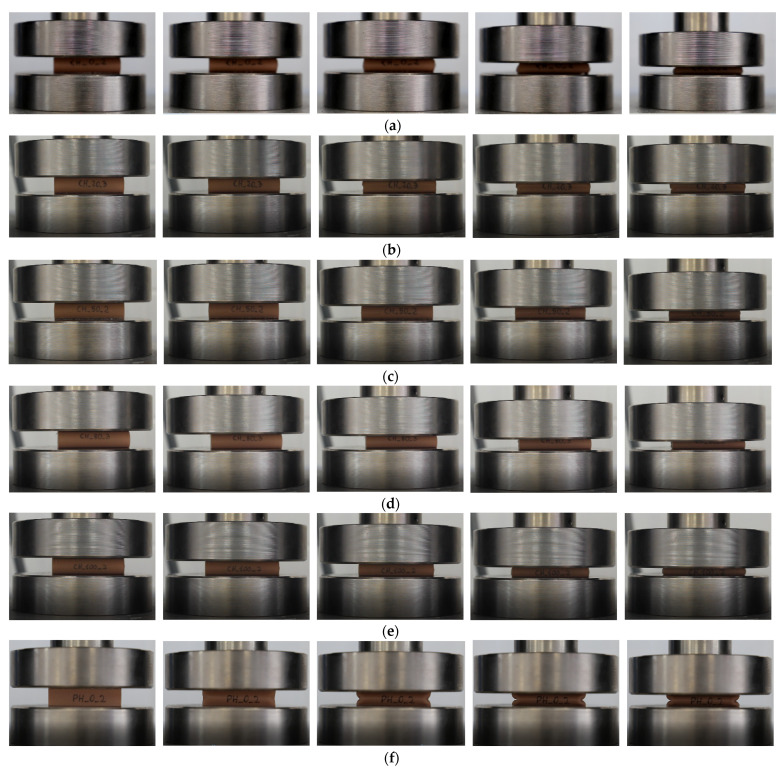

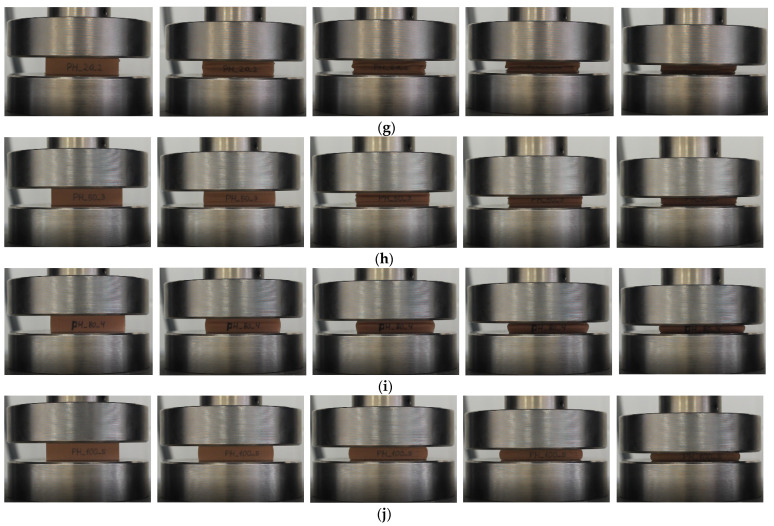
Test video with horizontal specimens: (**a**) CH 0%; (**b**) CH 20%; (**c**) CH 50%; (**d**) CH 80%; (**e**) CH 100%; (**f**) PH 0%; (**g**) PH 20%; (**h**) PH 50%; (**i**) PH 80%; (**j**) PH 100%.

In the case of the specimens in Figure A1 of Appendix B, which are horizontally oriented, it is observed that the specimens are fully compressed. Therefore, the combination of the horizontal printing orientation and the infill density could be applied to many products that have to absorb deformations due to their specific function [54].

**Figure A2:**
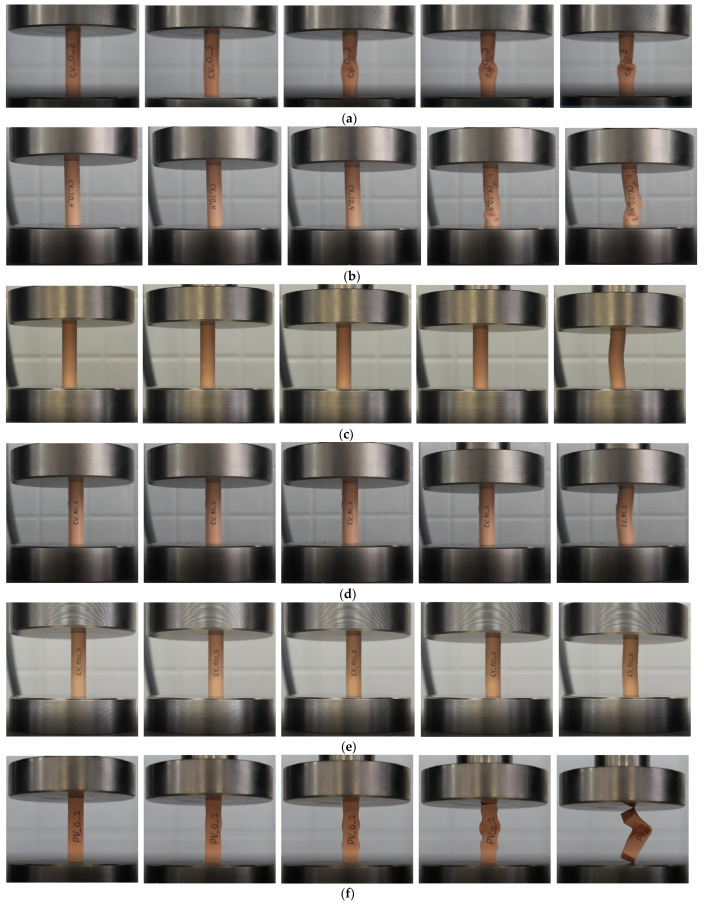

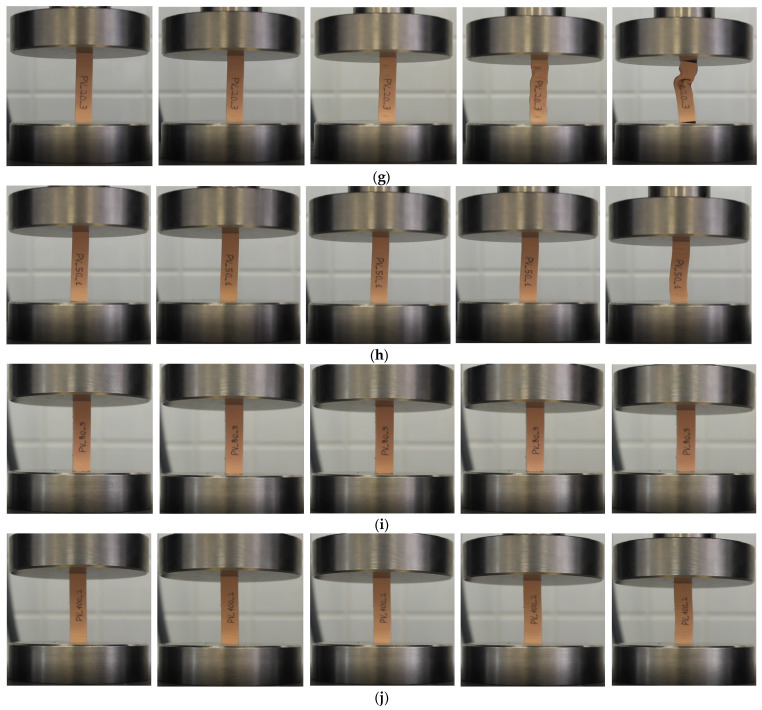
Test video with vertical specimens: (**a**) CV 0%; (**b**) CV 20%; (**c**) CV 50%; (**d**) CV 80%; (**e**) CV 100%; (**f**) PV 0%; (**g**) PV 20%; (**h**) PV 50%; (**i**) PV 80%; (**j**) PV 100%.

However, in the vertically oriented specimens in Figure A2 of Appendix B, the compression behavior is significantly different. In some examples, it is observed that in the central part there are zigzag folds that produce an increase in the compression distance, compressing the initial length to a large extent. On the other hand, in other specimens with such folds, the stiffness of the geometry does not allow for compression and the specimen bends. This causes warping of the geometry and even detachment of the test area. This behavior can be due to the different filling densities as well as the external walls that also influence the elastic behavior of the product.

**Figure 5:**
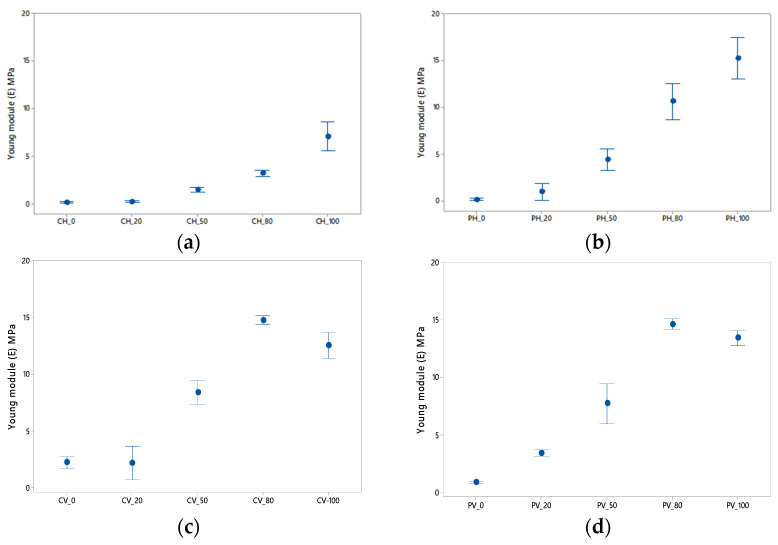
Elastic module according to the % of lightening: (**a**,**b**) horizontal orientation; (**c**,**d**) vertical orientation. (**a**,**c**) are cylindrical in shape and (**b**,**d**) are prismatic in shape.

Figure 5 shows the elastic modulus data obtained as a function of the infill percentage. A preliminary description of the results reveals that the elastic modulus increases with increasing density. In particular, it is interesting to discuss each case in detail. Thus, the horizontally tested specimens show slightly lower values of elastic modulus due to the printing properties. As mentioned above, these properties vary depending on each axis (longitudinal and transverse). In this case, because the specimen has been printed vertically, the structure experiences a slight increase in E value in this position. This is especially noticeable for the vertical specimens with low density (0%, 20% and 50%).

As for the results obtained horizontally, the specimen with prismatic geometry achieves higher values of the elastic modulus. This may be due to the fact that the prismatic section is more stable during the test, since the circular geometry specimen is supported on the generatrix of the cylinder. This phenomenon is not noticeable for the specimens with a lower filling density, but it can be observed that for PH_80 and PH_100 the values obtained are 300% higher than for CH_80 and CH_100.

On the other hand, if the specimens are compared in a vertical position, it can be seen how the values are similar with the particularity that the upward trend of E with respect to the density percentage is broken for specimens with 80% filler. One reason for the slight decrease in the specimens with higher density may be the accumulation of defects discussed in the previous section as a consequence of the specimen having more material.

### 3.3. Validation with CAE

After the simulation study, the data obtained show that the laboratory experiments and the simulation are generally similar, Figure 5. It must be taken into account that the simulation does not represent the manufacturing defects and therefore the results may be affected. However, due to the fact that five specimens of each type and density have been tested, the representation of these defects can be mitigated by taking into account the deviation of results analyzed in the previous section.

**Figure 6:**
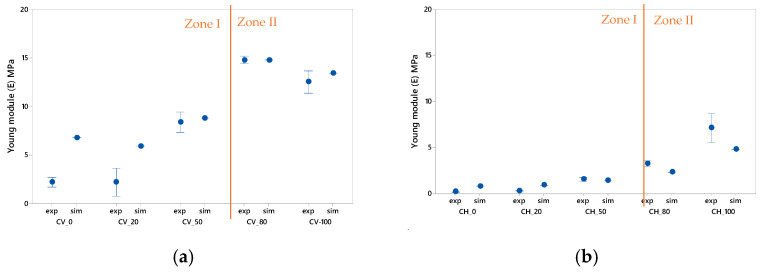

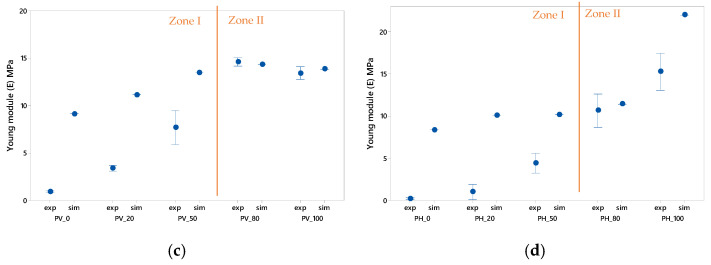
Comparative results of the experimental study (exp) and the simulation carried out (sim) for the yield stress: (**a**,**b**) cylindrical specimens; (**c**,**d**) horizontal specimens.

For a more in-depth analysis of the results obtained, the graphs in Figure 6 can be separated into two zones.

- Zone I. Includes the data for specimens with 0%, 20% and 50% infill density. For these specimens with a lower infill density, the simulation results show a high dispersion compared to the experimental data, especially for specimens with prismatic geometry.

The analysis of circular geometry specimens reveals for Figure 6a how for specimens CV_0 and CV_20 the simulation results are significantly higher than the experimental results and how they are outside the deviation range obtained. In Figure 6b, the described phenomenon is reduced and is not appreciable. This could be due to the fact that the horizontal tests have a larger contact area, which would allow for a better damping of the defects that could appear. Similarly, in Figure 6c,d, the results show a similar trend for prismatic specimens with medium and low densities. Specifically, for the specimens PV_0, PV_20 and PV-50, as well as their counterparts PH_0, PH_20 and PH-50, the results show that as the infill percentage increases, the experimental and simulation values converge.

The explanation of this phenomenon is that for reduced infill percentages, the amount of defects produced in layers and reinforcements has a greater influence during tensile tests, according to [49]. Appendix A shows the defects for each of the test specimens.

- Zone II. Includes the data for the specimens with 80% and 100% infill density. In these specimens with higher infill density, the simulation results show similar values to those obtained during the tests.

Figure 6a,b show how the simulation results are within the experimental range. On the other hand, Figure 6c,d reflect the same trend, except in the case of specimen PH_100, where the mean values obtained from the tests show results close to 15 MPa and the simulation shows a value around 30% higher.

In this area, despite the fact that there are also defects produced during the production of the specimens, it can be observed that their influence is not decisive because the fibers have a greater capacity to absorb tension when the density of the filling increases.

## 4. Experimental Verification: Case of Study of Design and 3D Printing by FFF of Elastic Keyboard

A flexible keyboard was designed to verify and evaluate the results obtained in the study. The designed keyboard was entirely manufactured by FFF with the exception of the electronic components, which were added later. Once manufactured, a compression test was performed.

It was designed based on the basic geometries established in the test tubes tested in the study. Moreover, the morphological and physiological characteristics of the human hand were taken into account.

There are various measurements of importance for the creation of manual elements, especially the length of the phalanges of the fingers. The dimensional variables of the product were determined with respect to DIN 33402 [55]. These variables focused on the ratio of hand and phalanx measurements to the 95th percentile given by the standard for keyboard design.

**Figure 7:**
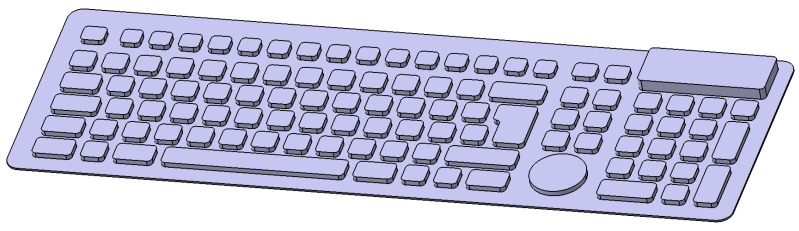
CAD image of keyboard design generated for additive manufacturing with TPU material.

Once the dimensions to be taken into account were defined, the configuration of the different functions of the keyboard was carried out according to the layout of the printed circuit used for the functional prototype. The inside of the keyboard must be prepared to contain the designed circuit. This is because the operation of the keyboard is based on the contact produced between the lines of the circuit when the conductive surface inside the key presses the printed circuit. Accordingly, each of the keys incorporates a lightened internal structure, which activates the key that is being pressed by compression. Therefore, each one of the keys is arranged according to the pattern of the printed circuit, obtaining the design shown in the Figure 7.

**Figure 8:**
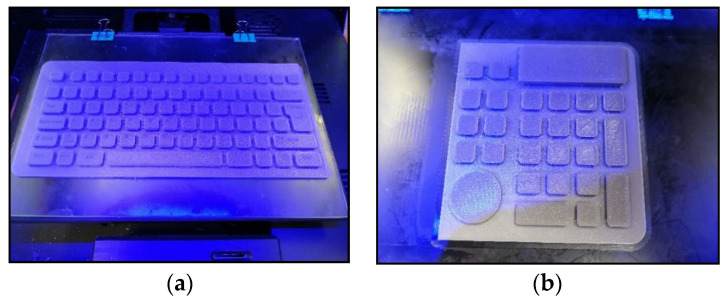
Images of the 3D printing process: (**a**) printing of the main body; (**b**) printing of the numeric keypad.

For the construction of the keyboard, Figure 7, the TPU material and the manufacturing parameters used in the elasticity study were used. The machine used was the 3D Creality CR-10 S5 Pro printer, because of its dimensions of 500 × 500 mm. Both parts, Figure 8a,b, were joined by means of post-processing.

Once the keyboard was printed, several compression tests were carried out in order to analyze the resistance and check that the results obtained in the elasticity study corresponded to the product designed.

The results obtained in the test, carried out on five different keys, are similar to the vertical cylindrical geometry with 0% filling, CV_0. The comparative results are shown in Table 5.

On the other hand, the structure of the keyboard, also tested on several different points, shows a greater increase in compressive strength because it is not lightened, as is the case with the prismatic specimen, PV_100. However, due to its reduced thickness, it remains elastic in its longitudinal direction and is therefore fully rollable.

**Figure 9:**
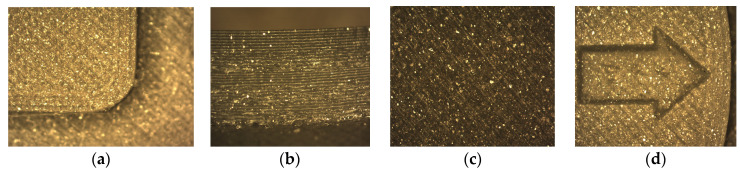
Optical microscopy images generated on the printed keyboard: (**a**) top view of key edge; (**b**) side view of key edge; (**c**) flat keyboard surface; (**d**) symbol engraving top view.

In an analysis carried out by optical microscopy, using the same apparatus as in the elasticity study, it can be seen that the finish obtained in the changes in relief and the surface present good quality, even better in the test tubes, Figure 9. This may be due to the impression height, which in this case is lower.

## 5. Conclusions

In this work, the evaluation of the compressive strength properties of additively manufactured TPU material specimens is presented. This work explores a field not studied so far, which focuses on the search for elasticity for the design of flexible custom products using flexible elements. The capacities of adapting to changes in design and creating elements with variable internal geometry offer an opportunity to develop flexible elements which can customize the level of flexibility of certain areas of the same element. Therefore, this line of research is key to the characterization of elastic parameters according to the internal structure of the product.

The procedure presented here for the customized design of elastic products offers great advantages in the field of new product design. Thanks to the design of internal structures with densities specifically conceived for the designed product, it opens up a new line of work that can be applied to different fields, such as consumer products or healthcare.

According to the results, it was observed that the variation of the infill density progressively varies the final elasticity of the product. Therefore, from a product design perspective, the force required to compress the geometry varies according to the infill density.

After analysis of the data, it was detected that the level of elasticity achieved for the density at 80% filler is lower than that at 100% solids. This classification of elasticity according to the internal printing properties has a clear potential to create customized products with different elasticities, even within the same product.

In addition, with this line of work it is possible to reduce the material used, the printing time and the weight of the product without varying the mechanical properties. It was observed that for a certain given hardness or elasticity it is possible to create products with lower weight and use of material. Accordingly, this finding is relevant for the design of parts with savings in costs and manufacturing time. For this last reason, future studies focused into more complex geometries and other internal structures are considered to be of interest.

On the other hand, compression tests have been performed at a constant speed, according to [42]. As a future line of research, the study of different velocities is proposed in order to observe the behavior of the samples with respect to these variations. Deepening the analysis of internal structures and densities customized to the specific purpose of the product is also considered to be of interest.

Regarding the potential application of TPU for AM, it is considered to be of great value for its elastic properties and ease of adaptation, particularly for highly ergonomic products. However, the limitations still existing in FFF technology with flexible materials keep open a field of research to work on topological properties with the aim of increasing the performance of additive manufacturing with TPU and the final quality of these products. This may lead to potential increased applicability in all types of sectors and products.

In short, the data obtained are considered to be of great value for subsequent applications to elastic or flexible products. In this way, the geometry of the product can be customized with respect to its specific function.

## Figures and Tables

**Table 1:** Main mechanical and physical properties of TPU, provided by Recreus.

Material	Filament Diameter (mm)	Density (g/cm^3^)	Tensile Strength (MPa)	Melting Temperature (K)	Elasticity (MPa)	Shore Hardness A
**TPU**	1.75	1	55	488	26	82

**Table 2:** Manufacturing process parameters defined for Filaflex^®^ 82A TPU filament.

Parameter	Recommended Value	Experimental Value
Layer height [mm]	0.08–0.3	0.1
Wall thickness [mm]	-	1
Printing speed [mm/s]	15–100	20
Flow [%]	-	100
Extrusion temperature [°C]	210–250	240
Retraction speed [mm/s]	25–160	25

**Table 3:** Filling percentages of Type A cylindrical and Type B prismatic specimens.

	0%	20%	50%	80%	100%
Type A	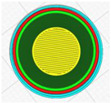	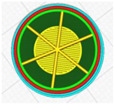	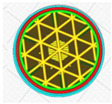	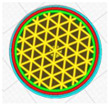	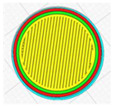
Type B	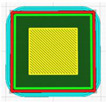	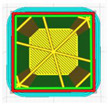	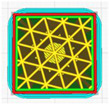	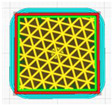	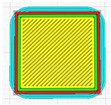

**Table 4:** Optimal manufacturing process parameters defined for Filaflex^®^ 82A TPU filament. P refers to prismatic specimens and C refers to circular specimens. H refers to horizontal compression specimens and V vertical compression specimens.

Horizontal Samples	Elastic Modulus E [MPa]	Standard Deviation	Vertical Samples	Elastic Modulus E [MPa]	Standard Deviation
PH-0	0.2	0.1	PV-0	0.9	0.1
PH-20	1.0	0.6	PV-20	3.4	0.2
PH-50	4.4	0.7	PV-50	7.7	1.1
PH-80	10.7	1.2	PV-80	14.7	0.3
PH-100	15.3	1,4	PV-100	13.4	0.4
CH-0	0.2	0.0	CV-0	2.2	0.3
CH-20	0.3	0.0	CV-20	2.2	0.9
CH-50	1.5	0.1	CV-50	8.4	0.7
CH-80	3.2	0.2	CV-80	14.8	0.3
CH-100	7.1	1.0	CV-100	12.5	0.7

**Table 5:** Results obtained from the compression test on the printed keyboard.

Element	Elastic Module [MPa]	Standard Deviation
TPU	26	-
CV_0	0.46	0.15
Structure	20.60	0.25
key	0.35	0.14

## Appendix A

**Table A1:** Optic microscopy of prismatic specimens.

	Base	Interlayer	Seam Line		Base	interlayer	Seam Line
PH_0				PV_0			
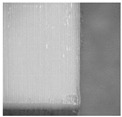
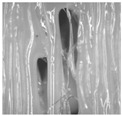
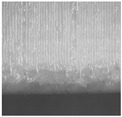
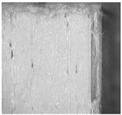
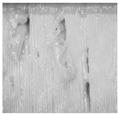
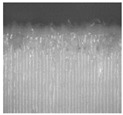
PH_20				PV_20			
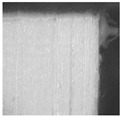
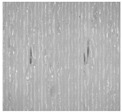
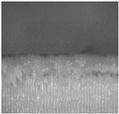
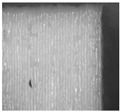
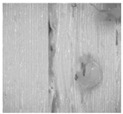
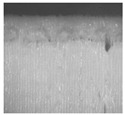
PH_50				PV_50			
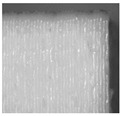
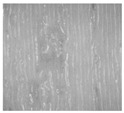
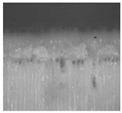
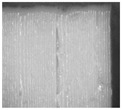
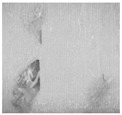
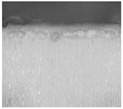
PH_80				PV_80			
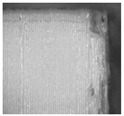
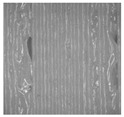
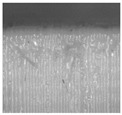
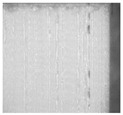
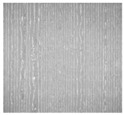
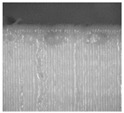
PH_100				PV_100			
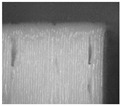
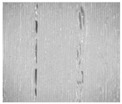
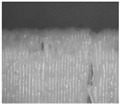
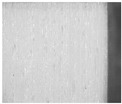
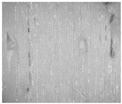
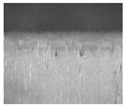

**Table A2:** Optic microscopy of cylinder specimens.

	Base	Interlayer	Seam Line		Base	Interlayer	Seam Line
CH_0				CV_0			
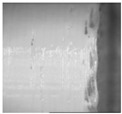
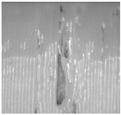
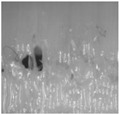
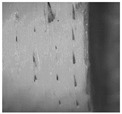
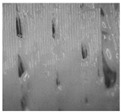
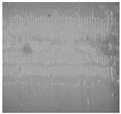
CH_20				CV_20			
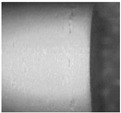
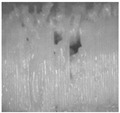
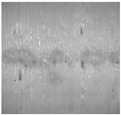
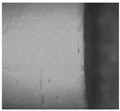
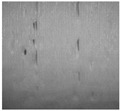
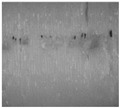
CH_50				CV_50			
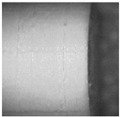
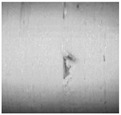
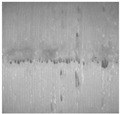
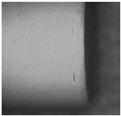
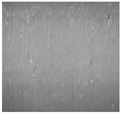
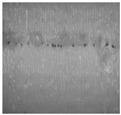
CH_80				CV_80			
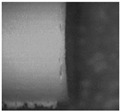
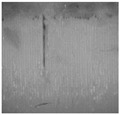
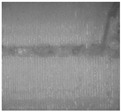
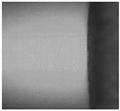
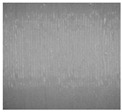
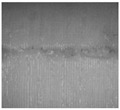
CH_100				CV_100			
